# Supplementary material for: Form and function of long-range vocalizations in a Neotropical fossorial rodent: the Anillaco Tuco-Tuco (Ctenomys sp.)
Source: PeerJ. 2016 Oct 11;4:e2559. doi: 10.7717/peerj.2559 (PMC5068419; doi:10.7717/peerj.2559)
Supplement: Supplemental Information 1 — Figure S1. Schematic representation of number of series and individual notes in the long-range vocalizations of the Anillaco Tuco-Tuco (Ctenomys sp.) recorded in the field. Each scheme is preceded by the number of individual, weight at time of the recording (in square brackets) and quantity of series without rec (dotted rectangle). Each scheme corresponds to a single recording. Wide gray boxes represent x number of series (i.e., dyad, triad or tetrad) and thin white boxes represent x number of individual note. [file peerj-04-2559-s001.pdf]

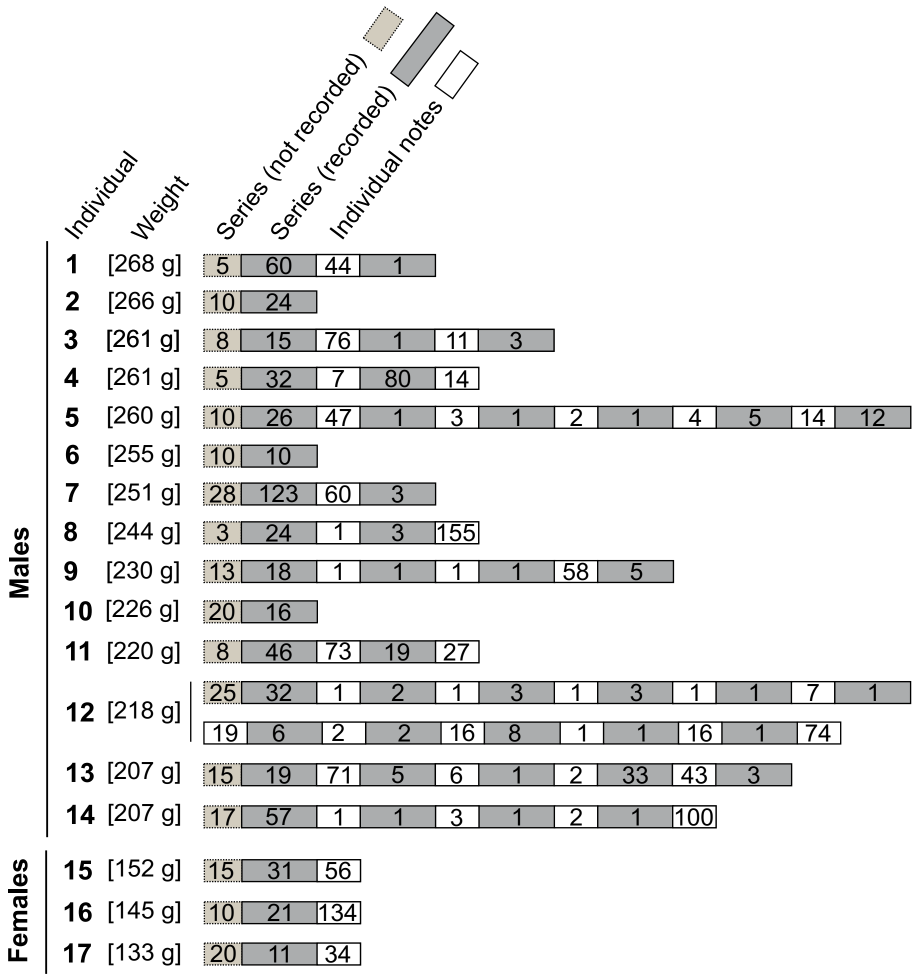

Series (not recorded)
Series (recorded)
Individual notes
Individual
Weight
Males
Females
1 [268 g] 5 60 44 1
2 [266 g] 10 24
3 [261 g] 8 15 76 1 11 3
4 [261 g] 5 32 7 80 14
5 [260 g] 10 26 47 1 3 1 2 1 4 5 14 12
6 [255 g] 10 10
7 [251 g] 28 123 60 3
8 [244 g] 3 24 1 3 155
9 [230 g] 13 18 1 1 1 1 58 5
10 [226 g] 20 16
11 [220 g] 8 46 73 19 27
12 [218 g] 25 32 1 2 1 3 1 3 1 1 7 1
19 6 2 2 16 8 1 1 16 1 74
13 [207 g] 15 19 71 5 6 1 2 33 43 3
14 [207 g] 17 57 1 1 3 1 2 1 100
15 [152 g] 15 31 56
16 [145 g] 10 21 134
17 [133 g] 20 11 34
