# Supplementary figures and images for: Form and function of long-range vocalizations in a Neotropical fossorial rodent: the Anillaco Tuco-Tuco (Ctenomys sp.)
Source: PeerJ. 2016 Oct 11;4:e2559. doi: 10.7717/peerj.2559 (PMC5068419; doi:10.7717/peerj.2559)

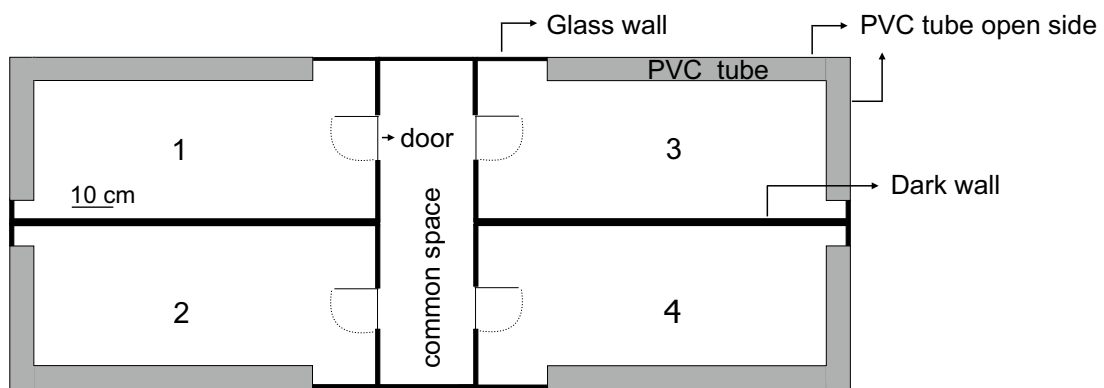

Supplement: Supplemental Information 2 — Figure S2. Schematic representation of the artificial burrow system where behavioral context of long-range vocalizations of the Anillaco Tuco-Tuco (Ctenomys sp.) was studied. [file peerj-04-2559-s002.pdf]

# Encounter with long-range vocalization

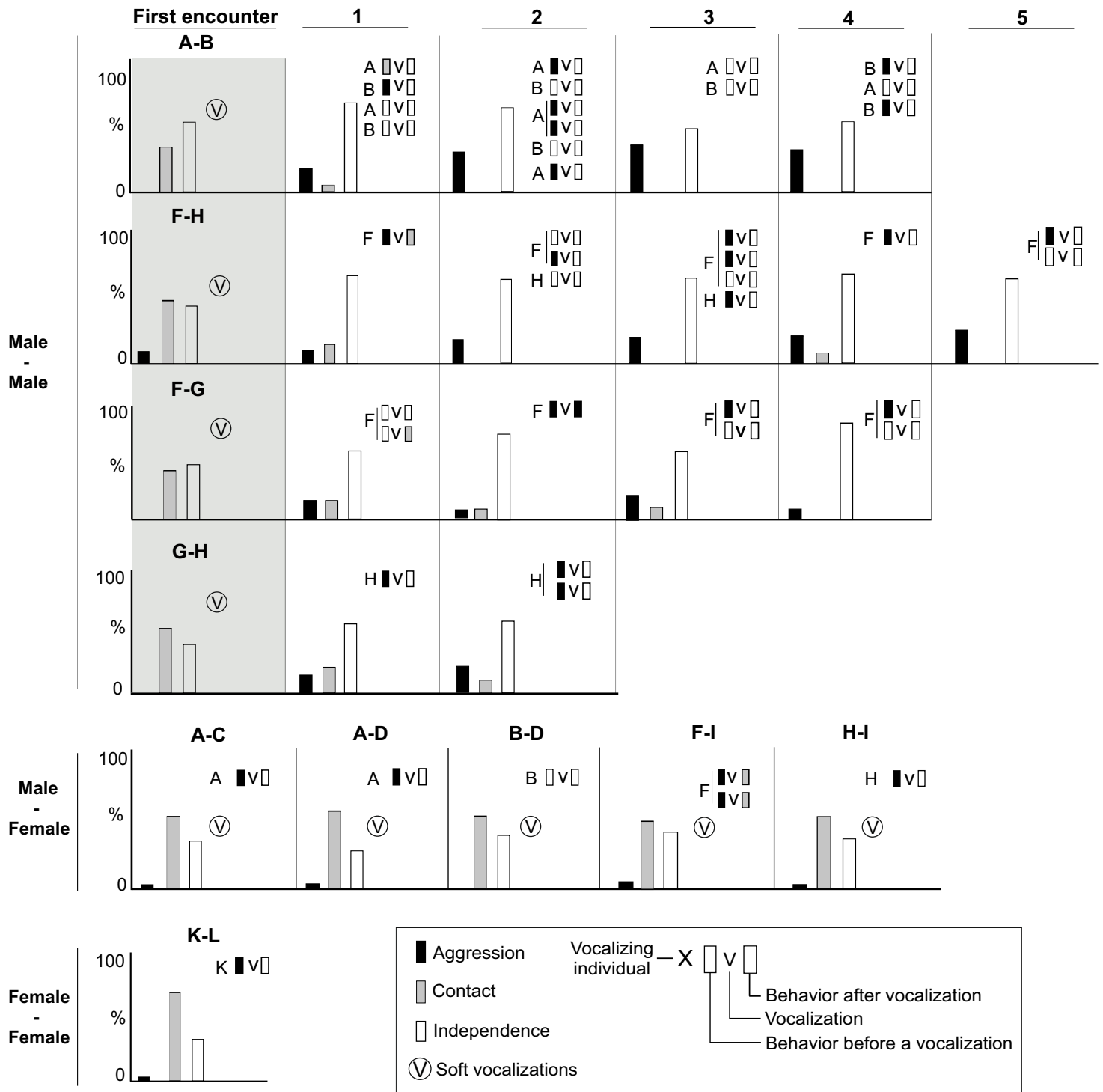

Supplement: Supplemental Information 3 — Figure S3. Behavior during staged vocal encounters of the Anillaco Tuco-Tuco (Ctenomys sp.) in captivity. Encounters with long-range vocalization (white background) and first male-male encounters with courtship vocalization (gray background). Bars indicate the percentage of occurrence of each behavior (Aggression, Contact and Independence) per encounter. [file peerj-04-2559-s003.pdf]
