# Supplementary material for: Form and function of long-range vocalizations in a Neotropical fossorial rodent: the Anillaco Tuco-Tuco (Ctenomys sp.)
Source: PeerJ. 2016 Oct 11;4:e2559. doi: 10.7717/peerj.2559 (PMC5068419; doi:10.7717/peerj.2559)
Supplement: Supplemental Information 4 — Table S1. Design of staged encounters between individuals of the Anillaco Tuco-Tuco (Ctenomys sp.) in laboratory experiments to assess the function of long-range vocalizations. Three types of encounters were performed: male-male, male-female and female-female. Capital letters indicate the identity of each individual used in each encounter type and numbers in parentheses indicats the number of staged encounters between the same individuals. [file peerj-04-2559-s004.docx]

|  | **Encounter type** | | |
| --- | --- | --- | --- |
|  | **Male-male** | **Male-female** | **Female-female** |
| **Individuals in each encounter**  **(n of encounters)** | A–B (5) | A–C (7) | C–D (3) |
|  | F–H (6) | A–D (5) | C–E (2) |
|  | F–G (5) | A–E (6) | D–E (2) |
|  | G–H (3) | B–C (6) | I–J (3) |
|  |  | B–D (5) | I–K (2) |
|  |  | B–E (3) | I–L (2) |
|  |  | F–J (3) | K–J (3) |
|  |  | F–I (3) | K–L (2) |
|  |  | F–K (2) | L–J (2) |
|  |  | G–J (3) |  |
|  |  | G–I (3) |  |
|  |  | G–K (2) |  |
|  |  | H–J (2) |  |
|  |  | H–I (3) |  |
|  |  | H–K (1) |  |
